# Maternal Thyroid Function During Pregnancy and Early Adolescent Regional Differences in Cerebral Gray Matter Morphology

**DOI:** 10.1210/clinem/dgaf349

**Published:** 2025-06-16

**Authors:** Tessa A Mulder, Ryan L Muetzel, Robin P Peeters, Henning Tiemeier, Tim I M Korevaar

**Affiliations:** Generation R Study Group, Erasmus University Medical Center, 3015 GD Rotterdam, The Netherlands; Department of Internal Medicine, Academic Center for Thyroid Diseases, Erasmus University Medical Center, 3015 GD Rotterdam, The Netherlands; Department of Child and Adolescent Psychiatry, Erasmus University Medical Center, 3015 GD Rotterdam, The Netherlands; Department of Child and Adolescent Psychiatry, Erasmus University Medical Center, 3015 GD Rotterdam, The Netherlands; Department of Radiology and Nuclear Medicine, Erasmus University Medical Center, 3015 GD Rotterdam, The Netherlands; Department of Internal Medicine, Academic Center for Thyroid Diseases, Erasmus University Medical Center, 3015 GD Rotterdam, The Netherlands; Department of Child and Adolescent Psychiatry, Erasmus University Medical Center, 3015 GD Rotterdam, The Netherlands; Department of Social and Behavioral Sciences, Harvard TH Chan School of Public Health, Boston, MA 02115, USA; Department of Internal Medicine, Academic Center for Thyroid Diseases, Erasmus University Medical Center, 3015 GD Rotterdam, The Netherlands; Division of Vascular Medicine and Pharmacology, Department of Internal Medicine, Erasmus University Medical Center, 3015 CN Rotterdam, The Netherlands

**Keywords:** thyroid, pregnancy, repeated measurements, offspring brain development, structural MRI

## Abstract

**Background:**

Thyroid hormone regulates fetal brain development. Both low and high maternal thyroid function during early pregnancy has been associated with smaller offspring total gray matter and cortex volume. However, it remains unknown whether regional gray matter differences underlie global brain morphology findings.

**Aim:**

To assess the association of gestational thyroid function with regional gray matter morphology through detailed vertex-wise analysis of cortical surface area and thickness and volumetric analyses of subcortical gray matter.

**Methods:**

We enrolled 2426 women of the population-based prospective cohort Generation R with TSH and/or free T4 (FT4) assessment before 18 weeks of gestation and offspring brain magnetic resonance imaging scans at age 10 and/or 14 years. We studied the association of gestational TSH, FT4, and (sub)clinical thyroid disease entities with local cortical surface area, thickness, and subcortical volumes.

**Results:**

There was an inverse J-shaped association of TSH with cortical surface area in the rostral middle frontal region (β [SE] for quadratic TSH: −0.005 [0.001] mm^2^, linear TSH 0.009 [0.004]). FT4 was not associated with cortical measures. Post hoc analyses revealed an inverse J-shaped association of TSH with gyrification in a similar region and children of hyperthyroid women had less gyrification in 3 cortical regions, mainly frontal (−0.082 [0.022], −0.077 [0.020], −0.069 [0.020]). Moreover, there was an inverse U-shaped association of FT4 with caudate volume (β [SE] for quadratic FT4: −0.004 [0.001] SD, linear FT4 0.010 [0.010]). TSH and FT4 were not associated with other subcortical volumes.

**Conclusion:**

Maternal thyroid function during early pregnancy is associated with offspring cerebral gray matter morphology in certain brain regions, specifically the frontal lobe. These findings expand on global brain morphology associations and support previous associations with behavioral outcomes.

Thyroid hormone is an important regulator of fetal brain development, including neuronal proliferation and differentiation (1). Animal studies showed that offspring born to mothers with low thyroxine have abnormal neuronal migration and cytoarchitecture in the somatosensory cortex and hippocampus (2, 3). In humans, the fetal thyroid gland is only functionally mature around 18 to 20 weeks of gestation; therefore, fetal brain thyroid hormone availability largely depends on the supply of maternal T4 during early pregnancy (4). These physiological principles have been translated to human epidemiological studies that showed that maternal thyroid (dys)function was associated with lower offspring IQ and neurodevelopmental disorders such as attention deficit hyperactivity disorder (ADHD) and autism (5-7). Moreover, our group has shown that both low and high maternal thyroid function during pregnancy is associated with less total gray matter and cortex volume in a large population-based cohort (6, 8). However, it remains unknown if maternal thyroid function is associated with regional differences in fetal brain development, as studies have focused on global brain measures. Identifying local differences can help to explain the association of maternal thyroid function during pregnancy with behavioral and cognitive outcomes and will allow for better characterization of thyroidal effects on brain development in future studies, including intervention trials.

A few studies have suggested region-specific effects of thyroid hormones on the brain. In 1 study, offspring of hypothyroid mothers had smaller hippocampal volumes and a thinner cortex in medial and mid-lateral regions, whereas the cortex was thicker in left superior regions and right inferior regions compared to controls (9). In another study, there were small but widespread regions of cortical thinning or thickening in children exposed to congenital hypothyroidism compared to controls (10). However, the interpretation of these studies is limited by a small size (n = 46 and 83), only including the hippocampus as subcortical structure, and not studying thyroid function on the continuum. The aim of this study was to assess the association of maternal thyroid function during pregnancy with regional offspring gray matter morphology.

## Methods

### Participants

This study was embedded in Generation R, a prospective population-based birth cohort (11). Eligible participants were pregnant women living in Rotterdam, the Netherlands, with an expected delivery date between April 2002 and January 2006. For the current study, mother–child pairs were included if maternal TSH or free T4 (FT4) was measured during pregnancy and a brain magnetic resonance imaging (MRI) scan was performed in the child at least once. Mother–child pairs were excluded in case of (treatment for) a maternal preexisting thyroid disorder, a twin pregnancy, or in vitro fertilization or if child brain MRI data quality was suboptimal or a major incidental finding was identified. We randomly excluded 1 of any sibling pair. The medical ethics committee of the Erasmus Medical Centre, Rotterdam, approved the study, and written informed consent was obtained from all adult participants and from both the parents of minors.

### Thyroid Measurements

TSH and FT4 concentrations were measured in serum samples obtained in early pregnancy (<18 weeks), stored at −80 °C, and measured using chemiluminescence assays (Vitros ECI; Ortho Clinical Diagnostics; Rochester, NY, USA). Hypothyroxinemia (normal TSH and low FT4), subclinical hypothyroidism (high TSH and normal FT4), subclinical hyperthyroidism (low TSH and normal FT4), and overt hyperthyroidism (low TSH and high FT4) were defined according to the 5th and the 95th population-based percentiles of thyroid peroxidase antibody (TPOAb) negative (<60 IU/mL) women. The latter was done according to the American Thyroid Association guidelines (12). The reference group consisted of women with a TSH and FT4 between the 5th and 95th percentile. Because of the limited number of women with hypothyroidism, we used the 10th and 90th percentile for FT4 and TSH for this entity, respectively. These cutoffs were chosen instead of the regular 2.5th and 97.5th percentiles for defining thyroid disease to optimize statistical power. The percentiles were defined in all women with available TSH and FT4 measurements without selection on availability of MRI outcomes.

### MRI Outcomes

In the current study, 2 data collection waves of brain MRI using the same scanner and protocol were used. Before scanning, children were familiarized with the scanning environment in a mock scanning session. MRI scans were obtained on a Discovery MR750w 3-T scanner (GE Healthcare, Milwaukee, WI, USA) with an 8-channel head coil. T1-weighted images were acquired using an inversion recovery prepared fast spoiled gradient recalled sequence. Cortical reconstruction and volumetric segmentation were performed with FreeSurfer, version 6.0.0 (13). The quality of FreeSurfer output was visually inspected, and all scans with insufficient quality were excluded from statistical analyses (14, 15). The number of cortical surface reconstruction defects was measured and used in sensitivity analyses. Local cortical thickness and surface area were derived from tessellated triangular surface meshes covering the cortex and smoothed with a full-width half max kernel of 10 mm. The local gyrification index in FreeSurfer was constructed using a 25 mm sphere applied to each vertex, which extracts the ratio between the amount of cortex buried within the sulcal folds as compared to the amount of cortex on the outer visible cortex (16). A large local gyrification index represents extensive folding of the cortex. Gyrification maps were smoothed with a 5 mm full-width half max kernel. In addition to local, vertex-wise cortical measures, subcortical volumes including the amygdala, hippocampus, thalamus, caudate, accumbens, cuneus, and putamen were studied. The volumes of left and right subcortical structures were averaged, as no lateralized effects were expected and to reduce the number of tests.

### Covariates

Potential confounders were selected a priori based on previous studies of the association of thyroid function with neurodevelopment outcomes (17) and biological plausibility. Information on maternal age at enrollment, national origin, parity (0, 1, or >1), smoking behavior (no smoking during pregnancy, smoked until pregnancy recognized, and continued smoking during pregnancy), and highest achieved education (high, intermediate, low) was obtained through questionnaires filled in during pregnancy. Body mass index was assessed at study enrollment and calculated as kg/m^2^. Midwives and hospital registries provided information on child sex. Gestational age at blood sampling was defined using ultrasonography. Child age at MRI scan was included as an independent predictor of MRI outcomes.

### Statistical Analyses

Prior to the analyses, outliers were excluded based on visual inspection of the data distribution (TSH n = 3 and FT4 n = 7).

First, linear mixed models were used to study the associations of TSH and FT4 with primary (ie, area and thickness) regional cortical brain metrics. Two MRI time points were studied simultaneously to increase power. Therefore, we included a random intercept for each child to account for clustered data. As we did not study change in MRI outcomes, no interaction term with child age at MRI scan was included. We used in-house developed software that runs regressions at each cortical vertex. In secondary analyses, we assessed thyroid disease entities as determinants of cortical vertex-wise outcomes. All models were adjusted for gestational age at blood sampling, maternal age, national origin, body mass index, smoking, parity, and education, and child sex and age at outcome assessment and inverse probability of attrition weights to correct for loss to follow-up and to increase generalizability to the whole cohort. Inverse probability weighting was based on aforementioned covariates, and weights were truncated at the 97.5th percentile (ie, extreme weights were reassigned to the 97.5th percentile value).

Nonlinearity was assessed by adding a quadratic term of TSH or FT4 to the model. TSH and FT4 were centered to their mean value to facilitate interpretation of the linear term. Effect modification by TPOAb status (above vs below 60 IU/mL) or by gestational age at blood sampling was assessed by adding product-interaction terms to the model with TSH or FT4. If this indicated effect modification, results were stratified by TPOAb status or by gestational age. We applied a cluster-wise correction using Monte Carlo simulation with a stringent cluster forming threshold (CFT) *P*-value of .001 to account for multiple testing. This CFT corresponds closely to a false-positive rate of 0.05 (18). An additional Bonferroni correction was applied for testing 2 hemispheres (*P* .025 cluster-wise). In post hoc analyses, more liberal CFTs of 0.005 and 0.01 were applied to assess whether results could be replicated in the other hemisphere and to rule out that laterality differences observed across hemispheres were an artifactual result of significance thresholding. To mitigate motion artifact effects, we adjusted our main findings for the number of defects in the cortical surface reconstruction in sensitivity analyses.

Next, we assessed associations of TSH and FT4 with subcortical volumes. All subcortical volumes were standardized to facilitate comparisons. Models were adjusted for intracranial volume. If adding a squared term for TSH or FT4 indicated nonlinearity, associations were visually inspected using a model with a restricted cubic spline with 3 knots. Subcortical analyses were corrected for multiple testing using the false discovery rate correction of Benjamini and Hochberg (19).

Missing covariate data was imputed 20 times with the multivariate imputation by chained equations method (20). TSH and FT4 concentrations, mean cortical thickness, and area and subcortical volumes were included as predictors for the imputation but were not imputed. Except for maternal national origin (1.3%), smoking (10%), and education (4%), missing data on covariates were less than 1%. All statistical analyses were performed with R statistical software version 4.2.1.

## Results

**Figure 1.**
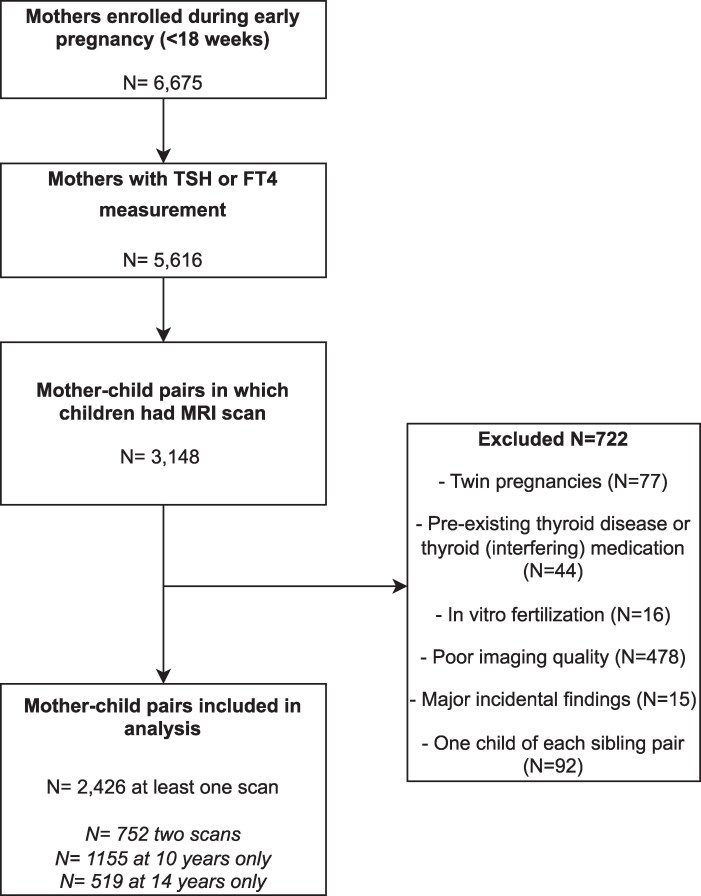
Flowchart of the study population.

The total study population comprised n = 2426 mother–child pairs, of which n = 752 children had MRI data on 2 time points (Fig. 1). Descriptive statistics of the study population are shown in Table 1. Mean (SD) gestational age at blood sampling was 13.4 (1.9) weeks. The median TSH and FT4 concentration were 1.3 mU/L and 14.8 pmol/L, respectively. Brain MRI scans were performed at mean age 10.2 and 13.9 years.

**Table 1.** Characteristics of the study population

Maternal age, years, mean (SD)	30.7 (4.7)
Maternal BMI, kg/m^2^, median [IQR]	23.4 [21.5, 26.2]
Parity, n (%)	
0	1469 (60.6)
1	710 (29.3)
≥2	247 (10.2)
Smoking, n (%)	
No smoking during pregnancy	1819 (75.0)
Stopped during pregnancy	228 (9.4)
Continued during pregnancy	379 (15.6)
Ethnicity, n (%)	
Dutch	1407 (58.0)
Moroccan	116 (4.8)
Turkish	134 (5.5)
Surinamese	197 (8.1)
European	193 (8.0)
Other Western	107 (4.4)
Other non-Western	272 (11.2)
Maternal education, n (%)	
High	1248 (51.4)
Intermediate	725 (29.9)
Low	453 (18.7)
Gestational age at blood draw, weeks, mean (SD)	13.4 (1.9)
TSH, mU/L, median [IQR]	1.3 [0.8, 2.0]
FT4, pmol/L, median [IQR]	14.8 [13.2, 16.7]
Child male sex, n (%)	1169 (48.2)
Child age at MRI first scan, years, mean (SD)	10.2 (0.6)
Child age at MRI second scan, years, mean (SD)	13.9 (0.6)

Total n = 2426 mother–child pairs. Data are shown after multiple imputation (see Methods section).

Abbreviations: BMI, body mass index; FT4, free T4; IQR, interquartile range; MRI, magnetic resonance imaging.

In nonresponse analyses, the TSH or FT4 concentration did not meaningfully differ between mother–child pairs with and without MRI data, although women included in the analyses were more often highly educated, had a higher mean age, were more often Dutch, and were less likely to smoke during pregnancy compared to those of mother–child pairs without MRI data (Supplementary Table S1) (21).

### Vertex-wise Cortical Outcomes

**Figure 2.**
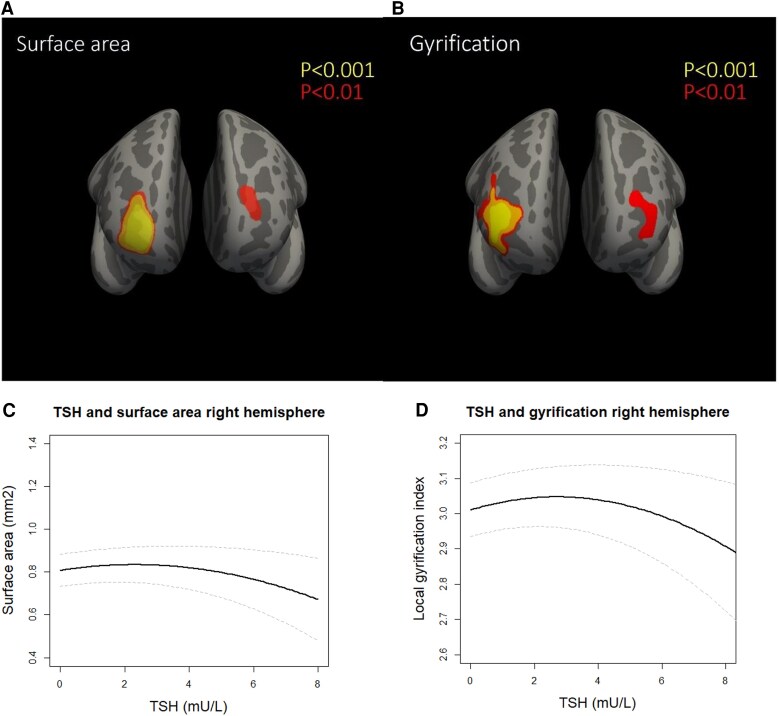
Association of maternal TSH during pregnancy with child cortical surface area (A and C) and gyrification (B and D). Surface area: n = 2393; β (SE) quadratic TSH = −0.005 (0.001); β (SE) linear TSH = 0.009 (0.004); annotation = rostral middle frontal region. Local gyrification index: n = 2378; β (SE) quadratic TSH = −0.005 (0.001); β (SE) linear TSH = 0.011 (0.004); annotation = rostral middle frontal region.

There was an inverse J-shaped association of TSH with surface area in the rostral middle frontal region of the right hemisphere (β [SE] for quadratic TSH: −0.005 [0.001] mm^2^, linear TSH 0.009 [0.004], Fig. 2). This association was also present in a similar region of the left hemisphere when a more liberal CFT *P*-value was used, albeit a smaller region than in the right hemisphere (Fig. 2). Results remained similar when adjusted for the number of surface reconstruction defects (data not shown). TSH was not associated with cortical thickness, and FT4 was not associated with cortical surface area or thickness. There was no effect modification by gestational age. TPOAb status modified the association of FT4 with surface area in some regions (*P* for interaction <.001), but this was not evident upon stratification (Supplementary Fig. S1, Supplementary Table S2) (21).

**Figure 3.**
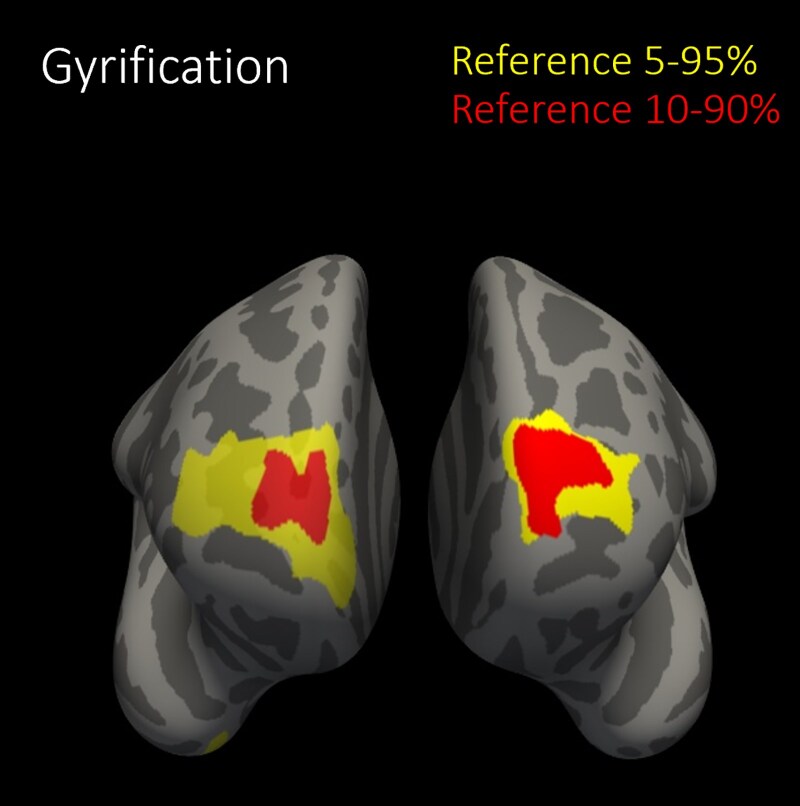
Association of maternal hyperthyroidism during pregnancy with child cortical gyrification. Reference group TSH and FT4 5% to 95%: n = 42/1943; average β (SE) −0.076 (0.021). Reference group TSH and FT4 10% to 90%: n = 75/1536; average β (SE) −0.057 (0.016). Annotations = rostral middle frontal, superior frontal, frontal pole, medial orbitofrontal, fusiform gyrus. Abbreviation: FT4, free T4.

Following our surface area results, we explored the local gyrification index as an outcome of maternal thyroid function in post hoc analyses. We observed similar results for gyrification in the same region as surface area (β [SE] for quadratic TSH: −0.005 [0.001], linear TSH 0.011 [0.004], Fig. 2). Moreover, children born to women with hyperthyroidism had less gyrification bilaterally in 3 clusters of vertices (average β [SE]: −0.076 [0.021], Fig. 3 and Supplementary Table S3) (21). In line with continuous TSH analyses, these regions were mostly located in the frontal lobe, although the regions were larger and the effect sizes were greater. Results remained similar when using the 10th and 90th percentile cut-offs to define hyperthyroidism (Fig. 3, Supplementary Table S3) (21). Findings for surface area of children prenatally exposed to maternal hyperthyroidism were in line with those for gyrification (ie, the same regions were found), but the associations with surface area did not remain when using the 10th and 90th percentile cut-offs, and effect sizes considerably attenuated (Supplementary Table S3) (21). There were no consistent findings for other thyroid disease entities or other cortical outcomes.

### Subcortical Outcomes

**Figure 4.**
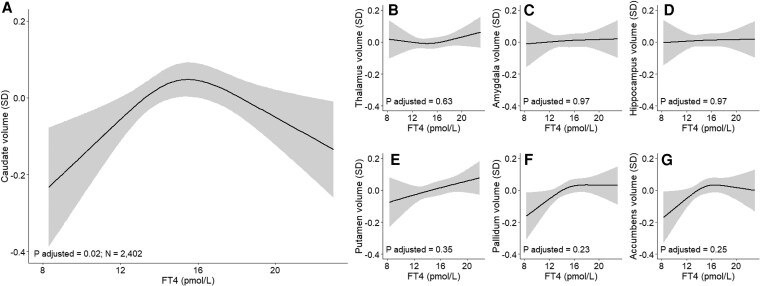
Associations of maternal FT4 during pregnancy with child subcortical gray matter volumes (A-G).

There was an inverse U-shaped association of FT4 with caudate volume (β [SE] for quadratic FT4: −0.004 [0.001] SD, linear FT4 0.010 [0.010], Fig. 4; Supplementary Table S4) (21) that remained after control for multiple testing. TSH or (sub)clinical thyroid disease entities were not associated with subcortical volumes (Supplementary Tables S4 and S5) (21). The association of TSH with putamen and caudate volume differed according to gestational age (*P* for interaction both .04 after multiple testing correction; Supplementary Table S6) (21). Stratified analyses by gestational age indicated an inverse U-shape of TSH with putamen and caudate volume around 9 weeks of gestation but not thereafter (Supplementary Fig. S2) (21).

## Discussion

In this population-based study, there was an inverse J-shaped association of maternal TSH during pregnancy with child cortical surface area and gyrification in the rostral middle frontal region but not with cortical thickness. FT4 was not associated with local differences in cortical morphology. Further, there was an inverse U-shaped association of FT4 with caudate volume in the subcortex.

To our knowledge, this is the first population-based study of maternal thyroid function and offspring regional cortical morphology. Several mechanisms can be considered for why we observed an inverse J-shaped association of maternal TSH with offspring cortical surface area and gyrification but not with thickness. Thickness and surface area are genetically and ontogenetically distinct features of the cortex, and their individual developmental trajectories are different from that of their product, namely cortical volume (22). In the current study, MRI outcomes were assessed during a time frame in which the downward trajectory of cortical thickness has started, while the decline in surface area starts somewhat later in adolescence and is less pronounced than that of thickness (23). Therefore, we carefully speculate that downstream effects of maternal thyroid disease in pregnancy on child cortical development might be characterized by decreased gray matter thickness initially but also less effective pruning in late childhood and adolescence, resulting in a dilution of the associations assessed in the current study. Alternatively, the power to detect differences in cortical thickness, a 1-dimensional construct, might have been lower compared to surface area. If the exposure of interest—maternal thyroid function in this study—affects multiple dimensions of cortical morphology, the effects on individual dimensions of cortical morphology might get amplified when a 2-dimensional construct like surface area is assessed, as previously hypothesized (24). In contrast to our study, associations between thyroid function and cortical thickness have been reported. Previous studies found locally thicker (superior and inferior) or thinner (medial and mid-lateral) cortex in children born to hypothyroid women (n = 22 cases) compared to children of euthyroid women (9) and small but widespread regions of cortical thinning or thickening in children with congenital hypothyroidism (n = 41 cases) compared to controls (10). These contrasting findings with our study might be partly due to differences in sample size and population, since these studies only included clinical cases. Moreover, as the preliminary findings by Lischinsky et al (9) only persisted with unsmoothed cortical thickness data and were not corrected for possible confounding, these findings may not be robust (18).

Our finding of less gyrification in the frontal cerebral cortex of children born to mothers with hyperthyroidism is not easily comparable to previous literature, as this cortical feature has, to the best of our knowledge, not been studied in relation to thyroid function in humans before. Gyrification and surface area are genetically and phenotypically related and thus cannot be considered completely independent (25). However, they only partially explain each other's relationship with cognition, which suggests that each contains unique information (25, 26). Interestingly, an animal study showed less cerebellar gyrification in offspring of type 3 deiodinase knockout mice (27). Also, local differences in cortical thickness and volume were reported in adult nonpregnant hyperthyroid patients compared to controls, which were mainly located in the frontal and temporal cortex (28). Several mechanisms can be considered for why our findings were most pronounced in the frontal cortex. Prior studies in animals and humans have shown that thyroid hormone receptors, deiodinases, and transporters are expressed in several brain structures, including the cerebral cortex (29-33), but there is no existing evidence of distinct frontal expression in the human cortex, nor are we aware of any evidence that the frontal cortex develops specifically in the gestational age range in which we measured thyroid function. Interestingly, the rostral middle frontal gyrus is part of the dorsolateral prefrontal cortex, which is involved in executive functions including attention, working memory, emotion regulation, and social cognition (34, 35), and this regional vulnerability could explain findings of maternal thyroid (dys)function with offspring ADHD, autism, and lower IQ (5-7).

We also observed an inverse U-shaped association of FT4 concentrations and caudate volume. The caudate is involved in, for example, planning of movement, memory, and emotion, and it has been linked to neurodevelopmental disorders including ADHD (36, 37), autism (38), and schizophrenia (39). Maternal thyroid dysfunction has been associated with ADHD and autism (7), although other studies did not find this for continuous thyroid function (40, 41). Moreover, the caudate has many connections with the frontal lobe cortex (42, 43). This is interesting, as we found associations of maternal thyroid function with offspring cortical surface area and gyrification in the frontal lobe. A study using fibroblasts of MCT8 patients and brain transcriptome data showed that the caudate nucleus is 1 of the brain regions in which the thyroid hormone transporter MCT8 is highly expressed (44). MCT8 deficiency is characterized by intellectual and motor disability due to cerebral hypothyroidism and peripheral thyrotoxicosis (45). Of note, MCT8 expression is also high in other subcortical regions for which we did not find associations (44). Nevertheless, we can carefully hypothesize that the caudate nucleus is involved in neurodevelopmental effects of thyroid (dys)function.

In our previous study, we showed that the association of maternal thyroid function during pregnancy with offspring cortical volume is most evident until 12 to 14 weeks gestation (8). However, we do not find evidence that the association of maternal thyroid function with offspring regional differences in gray matter morphology differs by gestational age except for some subcortical structures. These findings could be leveraged in future studies.

We were able to study the association of maternal thyroid function during pregnancy with regional differences in cortical morphology and subcortical volumes in early adolescence using a large, population-based cohort and utilizing state-of-the-art neuroimaging techniques. The large sample size allowed us to correct for many potential confounders. One limitation of this study is that TSH and FT4 were measured only once, which precluded studying changes in thyroid function during pregnancy in relation to brain development. Moreover, the number of women with (sub)clinical thyroid disease entities was limited. Furthermore, T3 concentrations were not measured in the Generation R cohort, and therefore these data are not available. Importantly, the majority of T3 in the fetal brain originates from T4 transport across the blood-brain barrier and subsequent conversion to T3. Thus, early fetal brain development depends almost entirely on maternal T4 and not T3.

In this population-based cohort study, maternal thyroid (dys)function was associated with cortical surface area and gyrification, mostly in the frontal area. These results indicate that maternal thyroid function during pregnancy is especially important for offspring cortical brain development in frontal regions. Future studies on maternal thyroid (dys)function during pregnancy leveraging even larger datasets for sufficiently powered mediation analyses should investigate the functional consequences of brain structural differences to explore the implications of our findings for cognition and behavioral problems.

## Data Availability

Some or all datasets generated during and/or analyzed during the current study are not publicly available but are available from the corresponding author on reasonable request. The datasets for this manuscript are not automatically publicly available due to legal and informed consent restrictions.
